# Is it renal colic or ruptured dissecting aneurysm of renal artery?: A case report

**DOI:** 10.1186/1757-1626-2-9398

**Published:** 2009-12-24

**Authors:** Sanjay Marwah, Sham Singla, Rajnish Kalra, Nisha Marwah, Shashi Pratap Singh

**Affiliations:** 1 Department of Surgery, Pt BDS Post Graduate Institute of Medical Sciences, Rohtak-124001, Haryana, India; 2 Department of Pathology, Pt BDS Post Graduate Institute of Medical Sciences, Rohtak-124001, Haryana, India

## Abstract

**Introduction:**

The dissecting aneurysm of renal artery is a form of renal artery occlusive disease that is infrequently recognized in the literature. However, when encountered, it is of great clinical significance because symptoms related to aneurysm are rarely seen and there is risk of its rupture.

**Case Presentation:**

The present case was a 30 year old Indian male, who presented with recurrent episodes of pain mimicking renal colic, which turned out to be a ruptured dissecting aneurysm of renal artery on exploration. The patient could not be salvaged due to delay in the diagnosis.

**Conclusion:**

This report highlights that rupture of renal artery aneurysm is a rare but potentially lethal clinical entity and should be considered as one of the differential diagnosis in patients with severe and persistent renal colicky type of pain in the absence of obvious findings on routine investigations.

## Introduction

Aneurysm of renal artery is a rare entity with an incidence of 0.01-0.09% reported on autopsy. However, with the advent of angiography and vascular computed tomography(CT)imaging, it has become clear that this entity is not so uncommon. The documented incidence on arteriograms is 0.73 to 0.97% [1,2]. Dissections of the renal artery may present either as an acute or chronic disease. Renal artery dissection may produce insignificant intramural hematoma, hemodynamically significant stenosis, or total occlusion, and, like other forms of renal artery occlusive disease, be completely silent and produce renal ischemia with associated renovascular hypertension or acute renal infarction [3]. The present article reports an unusual case of dissecting renal artery aneurysm with intra-peritoneal rupture that presented with clinical symptoms of renal colic.

## Case Presentation

A 30 year old male patient presented with three episodes of severe left renal colic during a period of one week and each time he had to come to emergency department. He was treated conservatively each time with ultrasound for kidneys being normal and urine showing numerous red blood cells. He was discharged on analgesics from the emergency with the advice to attend surgical outdoor for further investigations.

He presented once again in emergency during late night with acute pain abdomen and distension abdomen. He was looking extremely pale. His pulse was low volume with rate of 130/minute. His blood pressure was 86/50 mm of mercury. His abdomen was distended and tender on palpation. Shifting dullness was present on percussion and bowel sounds were absent on auscultation. The clinical findings were suggestive of some intra-abdominal catastrophe possibly acute hemorrhage. He was resuscitated with intravenous fluids, ionotrops and blood transfusions. Nasogastric aspiration revealed nonbilious aspirate. On urethral catheterization, 50 ml of hemorrhagic urine was drained. His hemoglobin was 5 gm % and routine urine examination revealed numerous red cells. His blood urea was 110 mg% and serum creatinine was 1.8 mg%. Portable X-ray chest and X-ray abdomen (sitting and lying) were done to look for any evidence of gut perforation (free air under the diaphragm) or intestinal obstruction (dilated gut loops with multiple air fluid levels). Only a few dilated gut loops were seen on X-ray abdomen. Portable ultrasound of the abdomen revealed free intra-peritoneal fluid and dilated gut loops. There was no perinephric fluid collection and both the kidneys appeared normal. Blood gas analysis showed metabolic acidosis and hypoxia.

In view of increasing abdominal distension and respiratory distress, tube drain was put in Morrison's pouch under local anesthesia on the bed side in area of maximum dullness in right flank as seen on abdominal percussion. It drained 2 litres of unclotted blood. Since there was continuous bleed as detected through constant trickle of blood in the drain, emergency laparotomy was performed with midline incision. On exploration, 2.5 liters of clotted and fresh blood was evacuated from the abdominal cavity. Liver, spleen, right kidney, stomach and intestines appeared to be normal. There was a large perinephric hematoma on left side of the midline that was gradually expanding. On opening the retroperitoneum on left of midline, the hematoma got disrupted and about 2 litres of blood came out with gush. The pulse and blood pressure of the patient became unrecordable and it was decided to pack the abdominal cavity as a life saving measure.

The patient was kept on ventilatory support in the post-operative period. MR angiography was planned but could not be performed due to poor condition of the patient. The re-exploration was done after 72 hrs when his blood pressure was partially stabilized with ionotropic support. At re-exploration, when sponges were removed, an arterial spurting was seen in left paraaortic region that was secured with vascular clamp. On careful inspection, the site of bleeding was found to be ruptured left renal artery aneurysm. The renal artery was secured proximal to the aneurysm and doubly ligated. The left kidney was removed. The right kidney on palpation appeared normal.

In post-operative period, the patient was again kept on ventilatory support and his condition remained critical. He had unstable vital signs and developed anuria. His blood urea and serum creatinine rose to 230 mg% and 3.8 mg% respectively requiring hemodialysis. However his condition kept on deteriorating and he expired on 3^rd ^post-operative day of second surgery.

**Figure 1:**
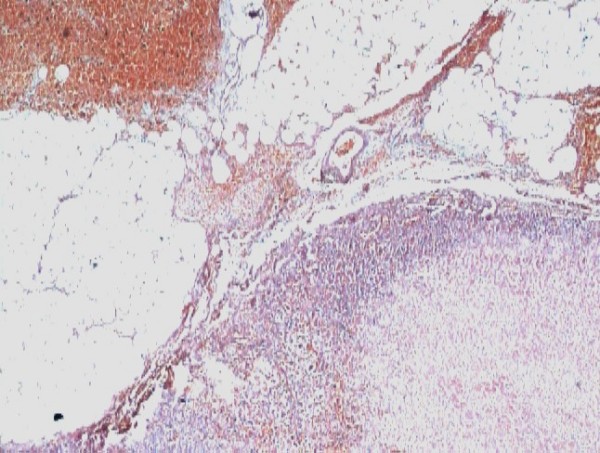
**Kidney showing infarction (H&E 100×)**.

**Figure 2:**
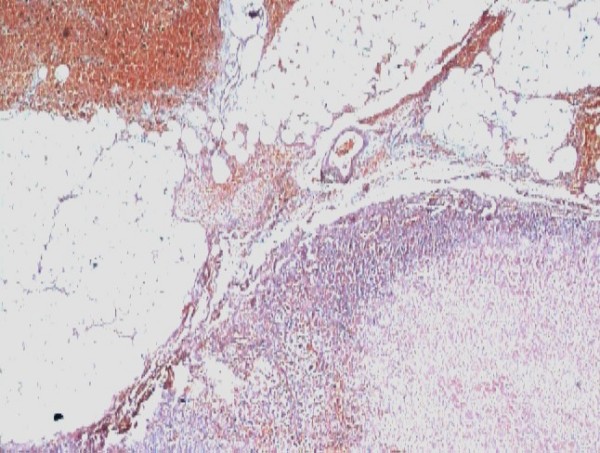
**Adrenal gland showing haemorrhage and necrosis with haemorrhage in periadrenal soft tissues (H&E 40×)**.

**Figure 3:**
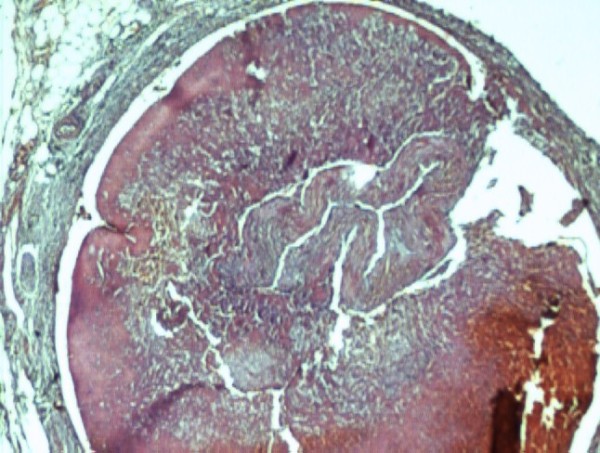
**Cross section of renal artery with extension of dissecting aneurysm with collapse of vessel (H&E 40×)**.

**Figure 4:**
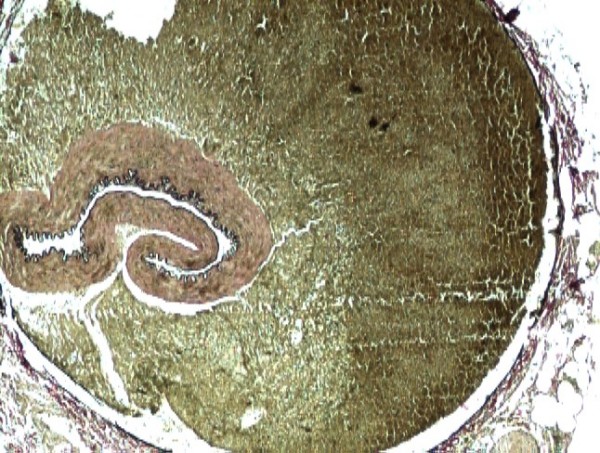
**Verhoff VG stain highlighting internal elastic lamina of collapsed renal artery (40×)**.

The biopsy of the specimen revealed massive areas of renal infarction (Fig. 1). There was haemorrhage in the periadrenal tissue (Fig. 2) with dissecting aneurysm involving the left renal artery and causing its collapse (Fig. 3, 4).

## Discussion

Renal artery aneurysm (RAA) is defined as a dilated segment of renal artery that exceeds twice the diameter of a normal renal artery. The first published report of RAA was in 1770 by Rouppe, who described the demise of a sailor who fell onto his right flank and the autopsy revealed a large false RAA with rupture [4]. Since that time, many more case reports and case series have provided most of the data on this rare pathological entity.

Predisposing factors for renal artery aneurysm include arteriosclerosis, fibromuscular dysplasia (FMD), congenital malformations of the kidney, renal angiomyolipoma, pregnancy and trauma [5]. Based on gross appearance, four types of renal artery aneurysm have been described: saccular, fusiform dilatation, aneurysmal dissection and intrarenal microaneurysms.

Renal artery aneurysmal dissection typically occurs in healthy men 40-60 years of age at a rate 10 times that in women. It is usually classified in two categories. In first category, blunt trauma leads to renal artery intimal stretching and tearing leading to dissection. In second category, the dissection may be spontaneous leading to pseudoaneurysm [6]. Although fibromuscular dysplasia is most frequently associated with renal artery dissection, but the etiology remains unclear in many cases as happened in the present case.

Most RAAs are asymptomatic and are found incidentally while investigating other intra-abdominal pathologies. Even symptomatic cases of renal artery aneurysm have non-specific presentations like haematuria, hypertension, flank pain, hydronephrosis and renal infarction.

Patients with RAAs caused by dissection are mostly asymptomatic but may present with colicky flank pain [3,5]. New or worsening pain may also be indicative of a rapidly expanding aneurysm or impending rupture. Patients with RAA rupture typically have signs and symptoms of an abdominal catastrophe and may be in frank shock [7]. Similar were the finding in the present case.

Although rare, but rupture is the most dreaded complication of RAA. It is reported that fewer than 3% of renal artery aneurysms rupture [8]. In another series, out of 252 RAAs encountered over 35 years, only three patients suffered from rupture [9]. Rupture of a renal artery aneurysm is an acute surgical event with a high mortality rate. However, the prognosis after rupture of RAA has improved in the last few decades. One review documented that the mortality rate dropped from 62% before 1949 to 6% after 1970 [10]. Another series reported a mortality rate of approximately 10% in males and non pregnant females [11]. Most authorities agree that pregnancy is associated with a significantly increased risk of rupture of renal artery aneurysm and it carries a high mortality rate. According to one report, renal artery rupture and its treatment resulted in death of the mother in 56% of the cases and death of the fetus in 78% of the cases [12].

In case of RAA rupture, plain film may show marginal calcifications of the aneurysm and distended bowel loops as a sign of retroperitoneal irritation. Ultrasound may demonstrate distended pelvis and perirenal collections. Ultrasound findings may be non-specific and similar to what is seen in renal infection, obstruction and urinoma. In present case, the only finding on ultrasound was distended bowel loops and free intra-peritoneal fluid. The possible cause for this free fluid was ruptured RAA leading to retroperitoneal hematoma that expanded rapidly and got burst into peritoneal cavity after eroding the posterior peritoneum.

When non-specific findings are seen with ultrasound, CT must be performed as soon as possible to make an accurate diagnosis. With the use of MDCT, the renal vasculature can be demonstrated and abnormalities are easier to depict. However these investigations are possible in hemodynamically stable patients only and could not be performed in the present case due to persistent shock.

If patient presents with ruptured RAA and is hemodynamically unstable, emergency surgery is required to control hemorrhage and prevent death. A midline approach with supraceliac aortic control is required because exposure of the renal vessels may be difficult in the presence of a large perinephric hematoma. The aortic cross clamp can be removed once the renal artery is controlled. In emergency with rupture of renal artery aneurysm, salvage of kidney may not be possible because of haemodynamic instability, and nephrectomy may be the option of choice, as it was in our case. However a case of acute retroperitoneal hemorrhage from a ruptured renal artery aneurysm has been reported in the literature that resolved spontaneously with resultant pseudoaneurysm formation that was ligated electively [13].

In a hemodynamically stable patient, renal salvage with renal artery reconstruction may be considered. Various modes of repair in elective surgery are repair of aneurysm, tangential excision with primary repair or patch angioplasty, excision with reconstruction using bypass and extracorporeal vascular reconstruction with autotransplantation [14].

## Conclusion

The aim of this report is to highlight that although rupture of RAA is well documented in the literature, but due to its rarity and nonspecific manifestations diagnosis is difficult leading to high mortality rate. In present case also presentation as recurrent acute flank pain with no clinical findings and investigations showing microscopic hematuria, it was considered as a routine case of renal colic. It subsequently lead to catastrophic hemorrhage and mortality because the rare possibility of ruptured RAA was not considered.

## Consent

Written informed consent was obtained from the family members of the patient for publication of this case report and any accompanying images. A copy of the written consent is available for review by the Editor-in-Chief of this journal.

## Competing interests

The authors declare that they have no competing interests.

## Authors' contributions

MS, SS, SSP managed and operated upon the patient. They also compiled the data, collected relevant data from the literature and analyzed it. KR and MN prepared and reported the microphotographs of the pathological specimen. They also compiled the data, collected relevant data from the literature and analyzed it.

Certified that all authors have read and approved the final manuscript.
